# A CpG 1018S/QS-21-Adjuvanted HBsAg Therapeutic Vaccine as a Novel Strategy Against HBV

**DOI:** 10.3390/vaccines13101014

**Published:** 2025-09-29

**Authors:** Zixuan Wang, Jing Wu, Xiaohan Meng, He Weng, Qiang Li, Lin Li, Zhenhao Ma, Sirong Bi, Qiuju Han, Huajun Zhao, Cunbao Liu, Deping Meng

**Affiliations:** 1 State Key Laboratory of Discovery and Utilization of Functional Components in Traditional Chinese Medicine, School of Pharmaceutical Sciences, Shandong University, Jinan 250012, China; 18345645559@163.com (Z.W.); w17660246037@163.com (J.W.); xiaohanmeng2001@163.com (X.M.); wobuxihuanwangy@163.com (H.W.); lq981228@163.com (Q.L.); 18054492327@163.com (L.L.); 18866497028@163.com (Z.M.); sirong_bi@163.com (S.B.); hanqiuju@sdu.edu.cn (Q.H.); zhaohuajun89@sdu.edu.cn (H.Z.); 2 Institute of Medical Biology, Chinese Academy of Medical Sciences and Peking Union Medical College, Kunming 650118, China; 3 The First Affiliated Hospital of Shandong First Medical University, Shandong Provincial Qianfoshan Hospital, Jinan 250014, China; 4 School of Clinical and Basic Medical Sciences, Shandong First Medical University and Shandong Academy of Medical Sciences, Jinan 250117, China

**Keywords:** chronic hepatitis B, therapeutic vaccine, adjuvant, CpG 1018, QS-21, immune tolerance, T cell exhaustion

## Abstract

Chronic hepatitis B virus (HBV) infection remains a major global health challenge, substantially contributing to liver-related morbidity and mortality. **Background/Objectives**: Developing therapeutic strategies that overcome immune tolerance and achieve functional cures is an urgent priority. **Methods**: In this study, we report a therapeutic vaccine comprising hepatitis B surface antigen (HBsAg) formulated with the dual adjuvant system CpG 1018S and QS-21. The immunogenicity and therapeutic efficacy of this vaccine were systematically evaluated in an rAAV8-HBV1.3-established chronic HBV mouse model. **Results**: The vaccine elicited a robust Th1-skewed immune response, characterized by elevated anti-HBs IgG2b titers and an increased IgG2b/IgG1 ratio. Notably, immunized mice showed markedly reduced circulating HBsAg levels. Mechanistically, the CpG 1018S and QS-21 adjuvant system enhanced dendritic cell activation, maturation, and antigen presentation, expanded HBV-specific CD4^+^ and CD8^+^ T cell populations, and attenuated the expression of the exhaustion markers TIM-3 and TIGIT. Additionally, immunized mice exhibited restored T cell polyfunctionality, with an increased secretion of effector cytokines, including TNF-α and IL-21. These responses collectively contributed to the reversal of T cell exhaustion and breakdown of immune tolerance, facilitating sustained viral suppression. **Conclusions**: Our findings demonstrate that the CpG 1018S/QS-21-adjuvanted vaccine induces potent humoral and cellular immunity against chronic HBV infection and represents a promising candidate for clinical chronic HBV (CHB) treatment.

## 1. Introduction

Hepatitis B virus (HBV) infection remains a pressing global public health challenge. An estimated 257 million people worldwide are chronically infected by this virus, placing them at risk for life-threatening complications, including cirrhosis, liver failure, and hepatocellular carcinoma [1,2]. A primary barrier to curing chronic HBV is viral persistence, which exhibits high tropism for hepatocytes. Within these cells, HBV establishes a stable mini-chromosome, the covalently closed circular DNA (cccDNA), that serves as a durable template for viral replication and transcription [3]. The long-term persistence of cccDNA, coupled with the integration of viral DNA into the host genome, leads to the continuous production of viral antigens, particularly hepatitis B surface antigen (HBsAg) [4]. This sustained high antigen load is a key driver of immune exhaustion, directly impairing the function of dendritic, B, and T cells, and fostering a tolerogenic microenvironment that prevents viral clearance [5].

Consequently, while current standard therapies—such as nucleos(t)ide analogues (NAs) and pegylated interferon-alpha (PEG-IFN-α)—can effectively suppress viral replication, they rarely achieve a functional cure, defined as sustained HBsAg loss [6,7,8], because these regimens do not eradicate cccDNA and, crucially, fail to reverse the underlying immune dysfunction. Therefore, restoring effective antiviral immune control is a fundamental therapeutic goal. In this context, therapeutic vaccines designed to overcome immune tolerance by re-engaging both humoral and cellular arms of the immune system have emerged as a promising strategy [9]. TG1050, a therapeutic vaccine based on a non-replicating adenoviral vector encoding multiple HBV antigens (S, core, polymerase), has demonstrated the ability to induce multifunctional HBV-specific T cell responses, reduce viral load and antigen levels, and exhibit favorable safety in preclinical and Phase Ib studies [10]. Currently, TG1050 shows particular potential when combined with TLR9 agonists in Phase II trials. Other therapeutic vaccines in clinical development include YIC (Phase III), Nasvac (Phase II/III), GS-4774 (Phase II), and several candidates in earlier-stage or preclinical development (e.g., HepTcell, AIC 649) [11,12,13]. However, these existing therapies (including the aforementioned vaccines) fail to achieve long-term virological control in the majority of chronic hepatitis B (CHB) patients. Considering HBV-associated antigen alone without efficient adjuvants is insufficient to break immune tolerance and induce HBV clearance, the rational design of new adjuvant systems capable of eliciting strong and well-orchestrated immune activation is central for developing a therapeutic HBV vaccine against HBV.

CpG oligodeoxynucleotides are well-characterized nucleic acid adjuvants that mimic microbial DNA, activate Toll-like receptor 9 (TLR9), and potently drive Th1-polarized immune responses with robust production of type I interferons and pro-inflammatory cytokines [14,15]. For instance, CpG 1018S, a synthetic TLR9 agonist, activates plasmacytoid dendritic cells (pDCs) and B cells, induces type I interferons and inflammatory mediators, and strongly promotes Th1-biased immunity. This activity is crucial for cytotoxic T cell (CTL) priming and effective antibody class switching. QS-21, a purified saponin derived from Quillaja saponaria (soapbark tree), enhances both Th1 and Th2 responses via multiple mechanisms, including the formation of antigen delivery complexes and markedly increased CTL responses and antibody titers [16,17].

Notably, CpG and QS-21 are key adjuvant components in licensed vaccines, such as Heplisav-B [18] and Shingrix [19]. Evidence from varicella zoster virus (VZV) vaccines indicates that the CpG/QS-21 combination elicits significantly stronger immune responses than either adjuvant alone, highlighting a synergistic effect [20]. CpG provides strong Th1 polarization and innate activation, while QS-21 broadens and amplifies both cellular and humoral responses, together creating an immune environment conducive to breaking tolerance. Supporting this, studies on herpes simplex virus (HSV) vaccines have shown that CpG/QS-21 combinations induce robust antigen-specific responses and confer protection against both HSV-1 and HSV-2 infection [21]. Although CpG adjuvants have been extensively studied in HBV vaccine research [22,23,24], the therapeutic potential of the CpG/QS-21 combination against chronic HBV infection remains largely unexplored. More critically, the precise immunological mechanisms by which this potent combination can reverse the deeply entrenched state of T cell exhaustion and break immune tolerance in an established chronic infection are completely unknown.

In this study, we investigate the immunogenicity and therapeutic efficacy of an HBsAg-based vaccine formulated with CpG 1018S and QS-21 in a chronic HBV mouse model established with rAAV8-HBV1.3 infection. By systematically assessing antibody subclass distribution, serum HBsAg clearance, dendritic cell activation, and T cell functionality, we delineate the immunomodulatory mechanisms underlying this adjuvant system and provide experimental evidence supporting its potential in therapeutic vaccine development for chronic HBV. Our study is the first to demonstrate that this specific adjuvant combination can effectively break tolerance in a persistent HBV model, primarily by revitalizing exhausted T cells and enhancing DC-mediated priming.

## 2. Materials and Methods

### 2.1. Animals and Reagents

C57BL/6 mice (6–8-week-old males) were purchased from Vital River Laboratories Animal Technology Co. in Beijing, China. All animals were housed under specific pathogen-free (SPF) conditions within Biosafety Level 2 (BSL-2) facilities located at the Model Animal Research Center of Shandong University. All procedures involving animals were strictly conducted in compliance with the Guide for the Care and Use of Laboratory Animals and adhered to the ethical standards set forth by the Shandong University Institutional Animal Care and Use Committee (IACUC), operating under an approved protocol (Authorization Number: 20023).

Reagents included the HBs peptide pool, covering the entire sequences of HBsAg (MESTTSGFLGPLLVL; SGFLGPLLVLQAGFF; PLLVLQAGFFLLTRI; QAGFFLLTRILTIPQ; LLTRILTIPQSLDSW; LTIPQSLDSWWTSLN; SLDSWWTSLNFLGGA; WTSLNFLGGAPTCPG; FLGGAPTCPGQNSQS; PTCPGQNSQSPTSNH; QNSQSPTSNHSPTSC; PTSNHSPTSCPPICP; SPTSCPPICPGYRWM; PPICPGYRWMCLRRF; GYRWMCLRRFIIFLF; CLRRFIIFLFILLLC; IIFLFILLLCLIFLL; ILLLCLIFLLVLLDY; LIFLLVLLDYQGMLP; VLLDYQGMLPVCPLL; QGMLPVCPLLPGTST; VCPLLPGTSTTSTGP; PGTSTTSTGPCKTCT; TSTGPCKTCTIPAQG; CKTCTIPAQGTSMFP; IPAQGTSMFPSCCCT; TSMFPSCCCTKPSDG; SCCCTKPSDGNCTCI; KPSDGNCTCIPIPSS; NCTCIPIPSSWAFAR; PIPSSWAFARFLWEW; WAFARFLWEWASVRF; FLWEWASVRFSWLSL; ASVRFSWLSLLVPFV; SWLSLLVPFVQWFAG; LVPFVQWFAGLSPTV; QWFAGLSPTVWLSVI; LSPTVWLSVIWMMWY; WLSVIWMMWYWGPSL; WMMWYWGPSLYNILS; WGPSLYNILSPFLPL; YNILSPFLPLLPIFF; PFLPLLPIFFCLWVY; FLPLLPIFFCLWVYI) for intracellular cytokine staining (ICS); hepatitis B surface antigen (HBsAg) expressed in Hansenula polymorpha (Dalian Hissen Bio-Pharm, Dalian, China); CpG 1018S (5′-TGACTGTGAACGTTCGAGATGA-3′) (Sangon Biotech, Shanghai, China); QS-21 (Alpha Diagnostic International Inc., San Antonio, TX, USA); HBsAg (Dalian Hissen Bio-Pharm, China), Alhydrogel 2% (alum adjuvant, Invivogen, San Diego, CA, USA); and the recombinant adeno-associated viral vector rAAV8-HBV1.3 carrying a 1.3-mer HBV genome (genotype D, serotype ayw) (PackGene Biotech, Guangzhou, China).

### 2.2. HBV-Carrier Mouse Model

A total of 60 C57BL/6 mice (6–8 weeks old) received a hydrodynamic tail vein injection of 0.6 × 10^10^ viral genomes (vg) of rAAV8-HBV1.3 recombinant virus diluted in saline (100 µL total volume). Mice exhibiting stable HBV-carrier status, confirmed by HBsAg > 500 ng/mL at 4–5 weeks post injection, were then randomly divided into three vaccination groups.

### 2.3. Vaccination

Mice in the experimental group (n = 6) received 100 µL intramuscular injections of the HBV recombinant protein vaccine, consisting of 2 µg of HBsAg and 50 µL of mixed CpG 1018S/QS-21 adjuvant (containing 10 µg of CpG 1018S and 5 µg of QS-21). Meanwhile, mice in the commercially available vaccine control group (n = 6) received injections of an equivalent volume (100 µL) of the alum-adjuvanted HBsAg (2 µg of HBsAg and 100 µg of Alhydrogel 2%). In contrast, mice in the blank control group (n = 6) received injections of an equivalent volume (100 µL) of phosphate-buffered saline (PBS, Servicebio, Wuhan, China). All mice underwent mandibular vein blood collection for serial monitoring. Finally, all injections were administered in a prime/boost regimen three times at two-week intervals.

### 2.4. Serum HBsAg and Anti-HBs IgG Subclass (IgG1/IgG2b) and ALT/AST Detection

Serum HBsAg levels were measured using commercial ELISA kits (HBsAg: Kehua Bio-Engineering, Shanghai, China) following the manufacturer’s protocols. Microplates (96-well) were coated overnight at 4 °C with HBsAg (5 μg/mL) to detect the anti-HBs IgG subclass. Subsequently, after blocking with 10% fetal bovine serum (FBS; ExCell Bio, Suzhou, China) for 2 h, 50 μL of serum samples was added and incubated at 37 °C for 1 h, followed by three washes. Then, horseradish peroxidase (HRP)-conjugated goat anti-mouse IgG/IgG1/IgG2b secondary antibodies (Cw Biotech, Beijing, China; diluted 1:5000) were added and incubated at 37 °C for 30 min, followed by three washes. Finally, 3,3′,5,5′-tetramethylbenzidine (TMB) substrate (Beyotime, Shanghai, China) was added for light-protected color development. The reaction was stopped with the stop solution, and absorbance was measured at 450 nm with 630 nm as the reference wavelength.

Serum ALT and AST levels were measured using commercially available kits (Nanjing Jiancheng Bioengineering Institute, Nanjing, China) according to the manufacturers’ instructions.

### 2.5. Mononuclear Cell (MNC) Isolation

Hepatic and splenic mononuclear cells (MNCs) were isolated using established protocols [25,26]. Liver tissues were mechanically dissociated through a 200 μm nylon mesh, followed by sequential centrifugation: initial clarification at 100× *g*, 2 min to remove hepatocytes; subsequent pelleting at 400× *g*, 5 min. The cell pellet was purified via 40% Percoll density gradient centrifugation at 800× *g*, 25 min (GE Healthcare, Uppsala, Sweden). Recovered MNCs underwent erythrocyte lysis and PBS washing. Splenic MNCs were processed identically after mechanical dissociation, followed by erythrocyte lysis. For intracellular cytokine staining, hepatic MNCs were resuspended in an RPMI-1640 medium (Gibco, Grand Island, NY, USA) supplemented with 10% FBS and 1% penicillin/streptomycin (NCM Biotech, Suzhou, China).

### 2.6. Flow Cytometry

MNCs were blocked with anti-CD16/CD32 (30 min, 4 °C), followed by surface staining with fluorophore-labeled monoclonal antibodies (30 min, 4 °C, dark). After three washes with phosphate-buffered saline (PBS), cells underwent fixation/permeabilization using the Foxp3/Transcription Factor Staining Buffer Set (eBioscience^TM^, Invitrogen, CA, USA) at room temperature (RT, 30 min, dark). Subsequent buffer washes preceded intracellular staining with the indicated antibodies (30 min, RT). Cell viability was assessed by staining with eFluor 506 fixable viability dye (eBioscience, #65-0866-14). The following fluorescently labeled antibodies were used: BUV737-anti-PD-L1 (eBioscience, #367-5982-82), BV650-anti-Ki67 (eBioscience, #416-5698-82), BV650-anti-CD62L (Biolegend, #104453), BV650-anti-CD86 (Biolegend, #105036), BV605-anti-mouse-CD127 (IL-7Rα) (Biolegend, #135041), BV605-anti-TNF-α (eBioscience, #406-7321-82), BV711-anti-CD103 (Biolegend, #121435), BV711-anti-mouse/human KLRG1 (MAFA) 2F1/KLRG1 (Biolegend, #138427), BV785-anti-mouse CD366 (Tim-3) RMT3-23 (Biolegend, #119725), BV785-anti-mouse-I-A/I-E (MHC-II) (Biolegend, #107645), BV421-anti-IFN-γ (Biolegend, #505830), FITC-anti-CD4 (eBioscience, #11-0041-85), FITC-anti-CD11b (Biolegend, #101206), PE-anti-mouse CD137 (4-1BB) (Biolegend, #106106), PE-anti-IL21 (eBioscience, #12-7213-82), PE-anti-Ly6C (Biolegend, #128007), PE/Cy7-CD3 Monoclonal Antibody (17A2) (eBioscience, #45-0031-82), PE/Cy7-anti-CD44 (Biolegend, #103030), PE/CF594-anti-CD8α (eBioscience, #61-0081-82), PE/CF594-anti-mouse TIGIT (Biolegend, #142110), PE/CF594-anti-IL-2 (Biolegend, #503840), Percp-Cy5.5-anti-F4/80 (Biolegend, #123128), Percp-Cy5.5-anti-CD3e (eBioscience, #45-0031-82), Percp-Cy5.5-anti-mouse CD11a (Biolegend, #101124), APC-Axl Monoclonal Antibody (MAXL8DS) (eBioscience, #17-1084-82), APC-anti-CD279 (PD-1) Monoclonal Antibody (J43) (eBioscience, #17-9985-82), APC-Cy7-anti-Ly6G/Ly6C (Gr-1) (eBioscience, #47-5931-82), APC-Cy7-anti-NK1.1 (Biolegend, #108752), AF700-anti-CD11c (eBioscience, #56-0114-82), and AF700-anti-CD8α (Biolegend, #100730).

### 2.7. Intracellular Cytokine Analysis

Hepatic MNCs were resuspended at 2 × 10^6^ cells/mL in complete RPMI-1640 (10% FBS) (Gibco, USA). Then, these cells were stimulated with an HBs peptide pool (10 μg/mL) in the presence of brefeldin A (BFA; 1 μg/mL, BioLegend, San Diego, CA, USA) for 16 h at 37 °C under 5% CO_2_. Following stimulation, cells were stained for surface markers, fixed/permeabilized (Cytofix/Cytoperm^TM^ Kit, BD), and incubated with the indicated antibodies. Data were acquired on a BD FACSymphony^TM^ A3 flow cytometer (BD Biosciences, San Jose, CA, USA). Data analysis was performed using FlowJo software (v10.8.1; FlowJo LLC, Ashland, OR, USA).

### 2.8. Statistical Analysis

All statistical analyses were conducted using GraphPad Prism 9.0 (GraphPad Software, La Jolla, CA, USA). Continuous variables were analyzed using unpaired Student’s t-test (two-group comparisons) or one-way ANOVA (multi-group comparisons) as appropriate. Data are expressed as the mean ± standard error of the mean (SEM). Statistical significance thresholds were defined as follows: * *p* < 0.05, ** *p* < 0.01, *** *p* < 0.001, **** *p* < 0.0001.

## 3. Results

### 3.1. CpG 1018S/QS-21-Adjuvanted HBsAg Elicits Robust Th1-Skewed Antibody Responses

**Figure 1:**
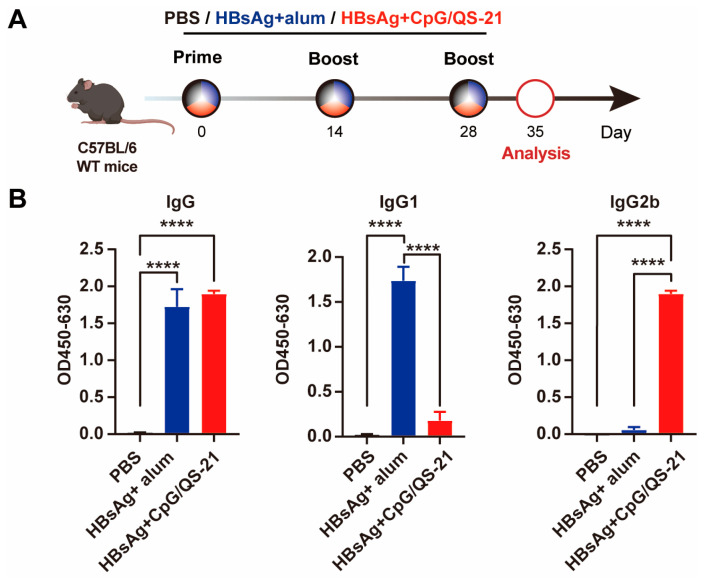
**CpG 1018S/QS-21-adjuvanted HBsAg induces high-level antibody responses in mice.** (**A**) C57BL/6J mice (n = 6/group, 6-week-old males) were intramuscularly vaccinated with HBsAg plus alum or HBsAg plus CpG 1018S/QS-21 vaccine at two-week intervals for three times, respectively, and control mice were administered with PBS. (**B**) C57BL/6J WT mice were vaccinated with CpG 1018S/QS-21-adjuvanted HBsAg vaccine or alum-adjuvanted HBsAg. Serum IgG, IgG1, and IgG2b antibody levels were measured using ELISA on day 7 after the third immunization. Error bars in data represent mean ± SEM. **** *p* < 0.0001 vs. controls.

To evaluate the immunogenicity of CpG 1018S/QS-21-adjuvanted HBsAg, wild-type (WT) C57BL/6J mice were immunized according to the scheduled protocol with PBS, HBsAg + alum vaccine, or HBsAg + CpG 1018S/QS-21 vaccine (Figure 1A). We measured serum levels of HBsAg-specific total IgG, IgG1, and IgG2b antibodies on day 7 after the third immunization with ELISA (Figure 1B). The HBsAg + CpG 1018S/QS-21 formulation induced total IgG titers comparable to the commercial HBV vaccine but with lower IgG1 responses, while significantly increasing anti-HBs IgG2b titers (1.7-fold vs. commercial vaccine; Figure 1B). The preferential elevation in IgG2b indicates a Th1-polarized humoral response driven by the CpG 1018S/QS-21 adjuvant system.

### 3.2. CpG 1018S/QS-21-Adjuvanted HBsAg Reduces Circulating HBsAg in HBV-Carrier Mice

**Figure 2:**
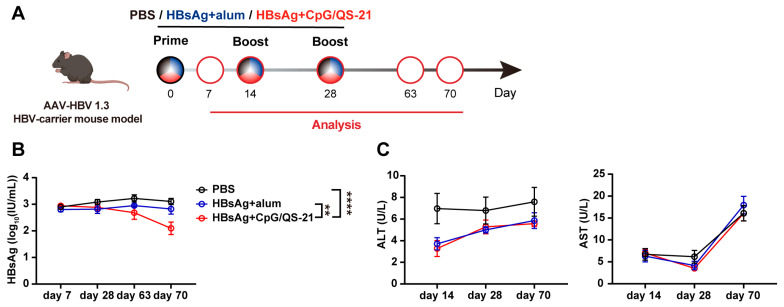
**CpG 1018S/QS-21-adjuvanted HBsAg reduces serum HBsAg in HBV-carrier mice.** (**A**) HBV-carrier mice were screened 6 weeks after intravenous injection of rAAV8-HBV1.3, followed by intramuscular vaccination with CpG 1018S/QS-21-adjuvanted HBsAg vaccine or alum-adjuvanted HBsAg in a two-week interval for three times, while control mice were administered with PBS. (**B**) Detection of serum HBsAg levels at days 7, 28, 63, and 70 post immunization with ELISA. (**C**) Serum ALT and AST levels were measured on days 14, 28, and 70 post immunization. Error bars in data represent mean ± SEM. ** *p* < 0.01, **** *p* < 0.0001 vs. controls.

We evaluated the therapeutic efficacy of CpG 1018S/QS-21-adjuvanted HBsAg in HBV-carrier mice. According to the scheduled protocol, HBV-carrier mice were immunized with PBS, HBsAg + alum vaccine, or HBsAg + CpG 1018S/QS-21 vaccine (Figure 2A). Serum collected on day 70 post immunization showed a significant decline in HBsAg exclusively in the HBsAg + CpG 1018S/QS-21 group, whereas PBS and the commercial vaccine produced no substantive change (Figure 2B). Simultaneously, serum ALT and AST levels were measured in mice. The HBsAg + CpG 1018S/QS-21 group showed no significant differences compared to both the PBS and commercial vaccine groups, and all values remained within normal ranges (Figure 2C). These data support the potential of CpG 1018S/QS-21-adjuvanted HBsAg as a therapeutic vaccine capable of lowering HBV antigenemia levels.

### 3.3. CpG 1018S/QS-21-Adjuvanted HBsAg Promotes Dendritic Cell Expansion and Activation

**Figure 3:**
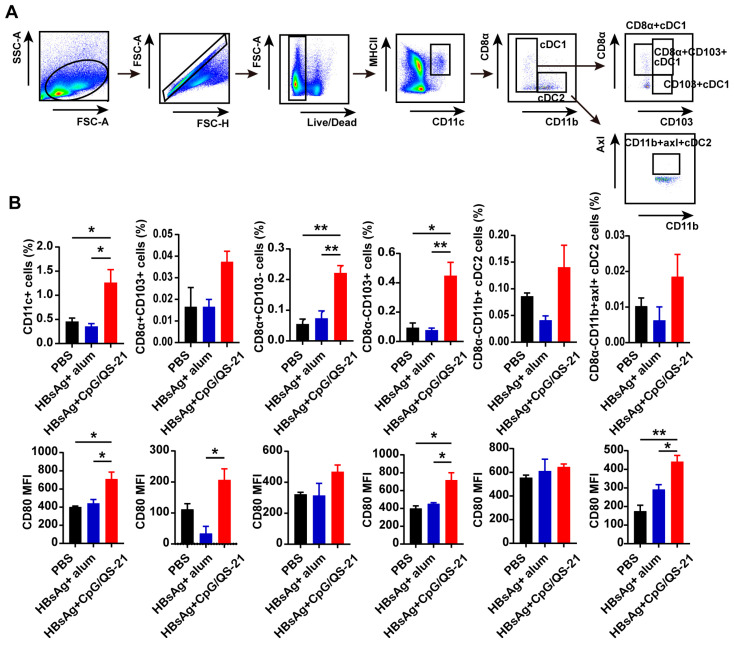
**CpG 1018S/QS-21-adjuvanted HBsAg expands splenic DC subsets and enhances activation.** MNCs from the liver and spleen were harvested 70 days after the treatment initiation. (**A**) Gating strategy for splenic DCs, CD8α^+^ CD103^+^ cDC1s, CD8α^+^ cDC1s, CD103^+^ cDC1s, cDC2s, and CD11b^+^ AXL^+^ cDC2s in the spleen. (**B**) The frequency and absolute numbers of DCs, CD8α^+^ CD103^+^ cDC1s, CD8α^+^ cDC1s, CD103^+^ cDC1s, cDC2s, and CD11b^+^ AXL^+^ cDC2s, and expression of CD80 on these cells in the spleen. Error bars in data represent mean ± SEM. * *p* < 0.05, ** *p* < 0.01 vs. controls.

Splenic dendritic cells (DCs) were analyzed via flow cytometry on day 70 post immunization to delineate innate mechanisms. Compared to PBS and the commercial vaccine, HBsAg + CpG 1018S/QS-21 significantly increased the frequencies of multiple DC subsets, including total DCs, cDC1s (CD8α^+^ and CD103^+^), cDC2s, and CD11b^+^ Axl^+^ cDC2s, accompanied by higher CD80 expression among these populations (Figure 3A,B). These findings indicate the CpG 1018S/QS-21 adjuvant enhances DC recruitment and maturation, providing a bridge between innate activation and adaptive priming against CHB infection.

### 3.4. CpG 1018S/QS-21-Adjuvanted HBsAg Alleviates T Cell Exhaustion and Enhances HBV-Specific T Cell Effector Functions

**Figure 4:**
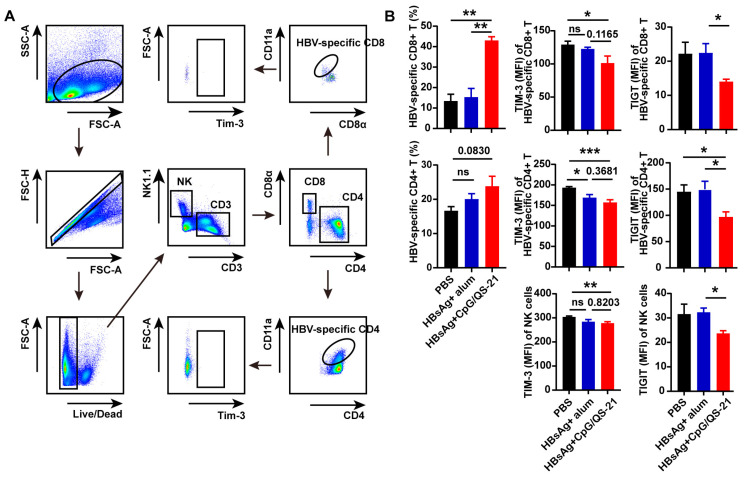
**CpG 1018S/QS-21-adjuvanted HBsAg reduces exhaustion markers and expands HBV-specific T cells in chronic infection.** (**A**) Gating strategy for HBV-specific CD4^+^ and CD8^+^ T cells and NK cells in the spleen. (**B**) The frequency and absolute numbers of HBV-specific CD4^+^ and CD8^+^ T cells, and NK cells, and expression of TIM-3 and TIGIT on these cells in the spleen. Error bars in data represent mean ± SEM. ns, not significant, * *p* < 0.05, ** *p* < 0.01, *** *p* < 0.001 vs. controls.

**Figure 5:**
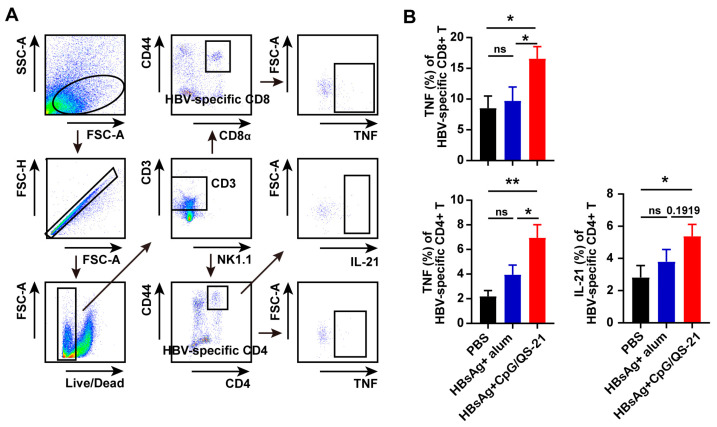
**CpG 1018S/QS-21-adjuvanted HBsAg enhances cytokine polyfunctionality in hepatic HBV-specific T cells.** (**A**) Gating strategy for HBV-specific CD4^+^ and CD8^+^ T cells in the liver. (**B**) Hepatic mononuclear cells were stimulated with HBsAg or preS1 antigen peptide pools (10 μg/mL) in the presence of Brefeldin A (BFA, 1 μg/mL) for 16 h in vitro. Proportions of HBV-specific CD4^+^ and CD8^+^ T cells producing IFN-γ, TNF-α, and IL-2 were analyzed via flow cytometry. The ability of HBV-specific CD4^+^ and CD8^+^ T cells to produce cytokines (TNF-α and IL-21) was analyzed by flow cytometry. Each pie chart represents the proportion of each cytokine population. Error bars in data represent mean ± SEM. ns, not significant, * *p* < 0.05, ** *p* < 0.01 vs. controls. MFI, mean fluorescence intensity.

Then, HBV-specific CD4^+^ and CD8^+^ T cells were evaluated via flow cytometry upon CpG 1018S/QS-21-adjuvanted HBsAg vaccination. The HBsAg + CpG 1018S/QS-21 group exhibited significantly increased proportions of HBV-specific CD4^+^ and CD8^+^ T cells [22], alongside markedly reduced TIM-3 and TIGIT expression on HBV-specific CD4^+^ and CD8^+^ T cells and NK cells (Figure 4), indicating the mitigation of T cell exhaustion. Functional profiling also revealed elevated cytokine production by intrahepatic HBV-specific T cells in the HBsAg + CpG 1018S/QS-21 group, including increased IL-21 in CD4^+^ T cells (2.5-fold vs. combined controls; ** *p* < 0.001) and TNF-α in CD8^+^ T cells (3.1-fold vs. combined controls; ** *p* < 0.001) (Figure 5). Together with DC activation (Figure 3) and reduced exhaustion (Figure 4), these results support a dual-pathway mechanism whereby CpG 1018S/QS-21 drives innate priming via DC maturation and subsequent adaptive restoration of adaptive immunity, thereby amplifying therapeutic efficacy against CHB infection, culminating in broad antiviral immunity enhancement.

## 4. Discussion

The fundamental pathological basis of chronic HBV infection is a dysfunctional virus-specific immune response, characterized by T cell exhaustion and immune tolerance [27,28,29]. Therefore, the primary therapeutic challenge lies in developing novel strategies capable of reversing T cell exhaustion and restoring antiviral immunity to achieve complete HBsAg clearance. A major limitation of current therapies is their inability to break immune tolerance, which accounts for the high relapse rates and the failure of most patients to attain a functional cure after treatment discontinuation. Multiple HBV therapeutic vaccine approaches have been developed for HBV treatment, including HepB-Eng (Engerix-B) and HepB-CpG (HEPLISAV-B), which comprise the HBsAg with alum adjuvants and CpG 1018, respectively. Considering the dual adjuvant CpG 1018S/QS-21 might show synergistic effects with a more potent and comprehensive humoral and cellular immune response than alum or CpG 1018 adjuvant alone, the CpG 1018S/QS-21-adjuvanted HBsAg therapeutic vaccine represents a promising candidate for clinical chronic HBV (CHB) treatment. Therefore, this study aimed to evaluate the CpG 1018S/QS-21-adjuvanted HBsAg therapeutic vaccine and its potential for treating chronic HBV infection. Our results demonstrate that the CpG 1018S/QS-21-adjuvanted HBsAg vaccine elicited stronger immune responses and achieved significant antigen clearance compared to a conventional commercial vaccine in a persistent HBV mouse model.

A key finding is that the CpG 1018S/QS-21-adjuvanted HBsAg vaccine induced a strongly Th1-polarized humoral immune response. The experimental group showed higher IgG2b antibody levels and an elevated IgG2b/IgG1 ratio (Figure 1B), indicative of Th1-biased immunity in mice. The immunologic mechanism of this combination is driven by the synergy between the TLR9 agonist CpG 1018S and QS-21. CpG potently activates antigen-presenting cells (APCs), stimulating secretion of cytokines such as IL-12 and TNF-α [24,30,31], which promote Th1 differentiation and drive B cell class switching toward the IgG2b subclass. This Th1-polarized response is closely associated with the activation of cytotoxic T lymphocytes (CTLs) and macrophages. Thus, the observed antibody isotype shift reflects enhanced cellular immunity, which is essential for clearing intracellular viruses and may represent a fundamental mechanism by which this adjuvanted vaccine overcomes immune tolerance.

Effective innate immune activation is critical for initiating adaptive immunity. Our data show that the CpG 1018S/QS-21 adjuvant combination significantly increased the abundance of multiple splenic DC subsets, including cDC1 and cDC2, and upregulated their expression of the activation marker CD80 (Figure 3). DC activation, maturation, and migration to lymph nodes are likely driven by the synergistic action of CpG (a TLR9 agonist) and QS-21. Given that cDC1 and cDC2 are essential for cross-priming CD8^+^ T cells and activating CD4^+^ T cells, respectively [32,33,34,35], this robust DC response provides critical signals for initiating potent adaptive immunity, effectively bridging innate and adaptive immune responses.

More importantly, the CpG 1018S/QS-21-adjuvanted HBsAg vaccine demonstrated substantial therapeutic efficacy in a chronic HBV infection model. It not only significantly reduced serum HBsAg levels (Figure 2B) but also directly reversed the exhausted phenotype of virus-specific T cells. We observed considerable expansion of HBV-specific CD4^+^ and CD8^+^ T cells, accompanied by a marked downregulation of the inhibitory receptor TIM-3 (Figure 4). Since TIM-3 is a well-established marker of T cell exhaustion, its reduced expression indicates the functional restoration of T cells [36,37,38]. Further analysis confirmed that these HBV-specific T cells produced higher levels of effector cytokines, including TNF-α and IL-21, and exhibited enhanced polyfunctionality (Figure 5). This functional recovery is critical for controlling viral replication and clearing infected cells.

Developing therapeutic vaccines for chronic HBV has long faced significant challenges, with numerous candidate vaccines like repurposed preventive vaccines (e.g., Engerix-B^®^) failing in clinical trials to substantially reduce HBsAg levels—the key indicator of functional cure. While capable of inducing antibody responses, these vaccines proved unable to reverse the T cell exhaustion and immune tolerance characteristics of chronic infection. This study adopts a breakthrough CpG/QS-21 adjuvant system, synergistically activating innate immunity to drive Th1-polarized responses, directly addressing the core bottleneck behind past failures. Its unique value becomes evident through systematic comparisons: Compared to DNA/viral vector vaccines, this protein subunit platform offers safety advantages, with the CpG/QS-21-induced multifunctional T cell responses (IFN-γ/TNF-α/IL-2) rivalling those of vector platforms without the anti-vector immunity risk [39]. In contrast to weakly adjuvanted vaccines like GS-4774, whose clinical failure stemmed from insufficient T cell activation [40,41], our strategy provides potent “danger signals”, effectively converting antigens into powerful immune attacks. Complementary to checkpoint inhibitor combination strategies, the vaccine itself fosters an inflammatory microenvironment through TLR9 (CpG)/QS-21 activation, capable of naturally counteracting immune suppression [42,43]. Strikingly, parallels exist with Shingrix^®^, a successful vaccine incorporating similar adjuvant principles: Shingrix^®^ employs the AS01B adjuvant (MPL + QS-21) to achieve >90% protection in immunosenescent populations [44]. Our vaccine similarly leverages QS-21 but replaces MPL with CpG 1018S (TLR9 agonist), uniquely enabling TLR9-driven immune restoration with potent CD8^+^ T cell priming and superior safety for HBV therapy. Furthermore, the CpG 1018S/QS-21-adjuvanted HBsAg therapeutic vaccine might induce synergistic effects with a more potent and durable humoral and cellular immune response against HBV than Heplisav-B^®^, which contains only the CpG 1018 adjuvant [45,46].

In summary, we propose that the therapeutic mechanism of the CpG 1018S/QS-21 adjuvant follows a coherent, closed-loop process: the potent composite adjuvant robustly activates dendritic cells, and then efficiently primes and expands HBV-specific T cells, as well as T cell polyfunctionality, resulting in effective viral control.

This study has certain limitations. First, considering we only demonstrated CpG 1018S/QS-21 (containing 10 µg of CpG 1018S and 5 µg of QS-21) as an efficient adjuvant for HBsAg therapeutic vaccine, the optimal dosage needs to be further evaluated. Second, since only AAV-transduced HBV-carrier mice were used in this study, the anti-HBV effect of the CpG 1018S/QS-21-adjuvanted HBsAg therapeutic vaccine should be further evaluated in other models, such as HBV transgenic mice and human liver chimeric mice supporting HBV infection. Additionally, the long-term durability and safety of the vaccine-induced immunity require extended observation. Future research will focus on translating this strategy into clinical applications and exploring its potential synergy with clinical antiviral drugs, such as entecavir, to ultimately provide a new therapeutic avenue for the functional cure of CHB.

## 5. Conclusions

The CpG 1018S/QS-21 adjuvant system effectively breaks immune tolerance in CHB infection. Its mechanism involves synergistic activation of dendritic cells to initiate a potent Th1-polarized response, which subsequently reverses virus-specific T cell exhaustion and restores T cell polyfunctionality, ultimately mediating effective viral control. This adjuvant combination represents a highly promising novel strategy for developing therapeutic vaccines against CHB.

## Data Availability

All data used during the study are available from the corresponding author upon request.
